# Commentary: Building a better mouse trap: Refining the minimally invasive giant paraesophageal hernia repair

**DOI:** 10.1016/j.xjtc.2021.08.029

**Published:** 2021-08-21

**Authors:** Siva Raja

**Affiliations:** Department of Thoracic and Cardiovascular Surgery, Heart and Vascular Institute, Cleveland Clinic, Cleveland, Ohio

**Figure:**
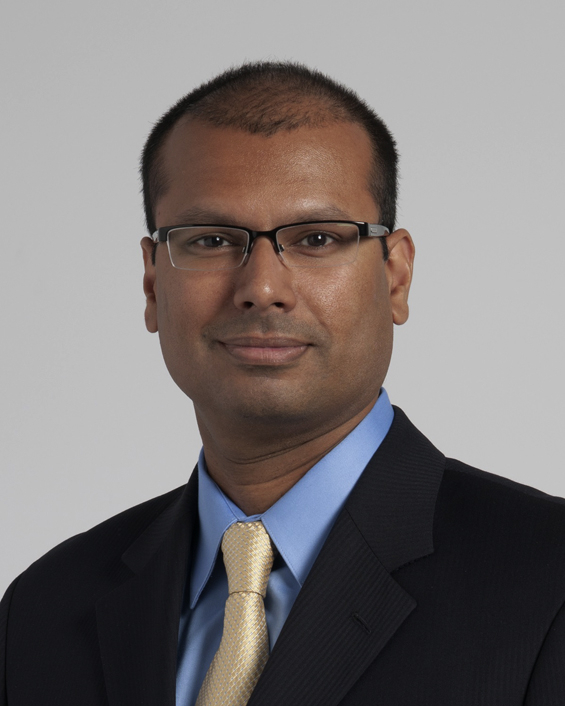
Siva Raja, MD, PhD, FACS

**Central Message**

Repair of giant paraesophageal hernia is complex and requires athoughtful and rigorous technique to optimize outcomes.

See Article page 497.

Giant paraesophageal hernia repair is becoming a commonly performed foregut operation. The use of minimally invasive techniques has revolutionized the operation, reducing morbidity and mortality.^1^ However, the use of minimally invasive techniques has not decreased the risk of recurrence of these hernia over open repair. While more contemporary series^2^ show low recurrence rates, they also lack long-term follow-up. In this issue, the Alicuban and colleagues^3^ from the University of Pittsburgh describe their technique for a minimally invasive repair of giant paraesophageal hernia. The technique highlights several of the important elements that are crucial to a robust repair, such as complete reduction of the hernia sac, preservation of the fascia on the crural pillars, extensive mediastinal mobilization, and appropriate evaluation of adequate esophageal length. The authors also provide excellent insights into the nuances of their technique, which are intended to address potential mechanisms of failure in this operation.

In my opinion, any conversation about giant paraesophageal hernia repair must also include (1) a consideration of shortened esophagus and (2) long-term hernia recurrence. Evaluation of intra-abdominal esophageal length is crucial, as the best protective mechanism against recurrence is a tension-free repair. In this Pittsburgh technique, the authors create a 3-cm length of intra-abdominal neoesophagus via a wedge gastroplasty. While many call it a Collis gastroplasty, a wedge gastroplasty does not truly create the cylindrical shape within the neoesophagus that is the hallmark of a Collis gastroplasty.^4^ Is it close enough? Is it effective enough? Is it long enough? These questions are not well understood yet, but the benefit of lengthening is undeniable in the patient with shortened esophagus.

The second issue at hand is that of recurrence. In the Pittsburgh series^5^ of 662 patients published in 2010, the recurrence rate was 15.7% at 22 months and a reoperation rate of 3.2% at 25 months. While this appears to be a low rate of recurrence at first glance, this rate at about 2 years foreshadows a much greater rate of recurrence at 10 years! It is here that their technique of placing the wrap within the vagal nerves is curious. They do so to decrease the rate of wrap slippage. While this may very well be the case, it is not likely to decrease the rate of recurrent hernia. In that case, one would suspect that the rate of vagal nerve damage would be much greater during the redo operation. It is for this reason that most of us will include the vagal nerves within the wrap. We hope for the best but plan for the worst.

In the world of paraesophageal hernia repair, we are constantly making changes to our technique to improve outcomes and decrease recurrence. It is important to consider that there is likely to be more than one successful technique. Foregut surgery is a balancing act of controlling symptoms while managing side effects. As it stands, we continue to decrease morbidity, but patients still suffer recurrence. This mechanical problem is not likely to be solved with a pill. As such, it is important to strive to make this mouse trap better so that future surgeons can build a better one on the shoulders of our successes.
